# Supplementary material for: Generation of hiPSC-Derived Skeletal Muscle Cells: Exploiting the Potential of Skeletal Muscle-Derived hiPSCs
Source: Biomedicines. 2022 May 23;10(5):1204. doi: 10.3390/biomedicines10051204 (PMC9138862; doi:10.3390/biomedicines10051204)
Supplement: Supplementary file 1 [file biomedicines-10-01204-s001.zip › biomedicines-1703149-supplementary.pdf]

SUPPORTING INFORMATION

Generation of hiPSC-derived skeletal muscle cells: exploiting the potential of skeletal muscle-derived hiPSCs

MDPI biomedicines (Research Article)

Special Issue: The Promise of Induced Pluripotent Stem Cells in the Biomedical Research

Eric Metzler [1,2,*], Helena Escobar [1,2], Daniele Yumi Sunaga-Franze [1,3], Sascha Sauer [1,3], Sebastian Diecke [1,4] and Simone Spuler [1,2,5,*]

[1] Max-Delbrück-Center for Molecular Medicine in the Helmholtz Association (MDC), Robert-Rössle-Str. 10, 13125 Berlin, Germany; helena.escobar@charite.de (H.E.); daniele.franze@mdc-berlin.de (D.Y.S.-F.); sascha.sauer@mdc-berlin.de (S.S.); sebastian.diecke@mdc-berlin.de (S.D.)

[2] Experimental and Clinical Research Center, a Cooperation between the Max-Delbrück-Center for Molecular Medicine in the Helmholtz Association and Charité—Universitätsmedizin Berlin, Lindenberger Weg 80, 13125 Berlin, Germany

[3] Max-Delbrück-Center for Molecular Medicine in the Helmholtz Association (MDC), Genomics Platform, Hannoversche Straße 28, 10115 Berlin, Germany

[4] Max-Delbrück-Center for Molecular Medicine in the Helmholtz Association (MDC), Pluripotent Stem Cells Platform, Robert-Rössle-Str. 10, 13125 Berlin, Germany

[5] Charité—Universitätsmedizin Berlin, Corporate Member of Freie Universität Berlin and Humboldt-Universität zu Berlin, Experimental and Clinical Research Center, Lindenberger Weg 80, 13125 Berlin, Germany

* Correspondence: eric.metzler@mdc-berlin.de (E.M.); simone.spuler@charite.de (S.S.)

Figure S1

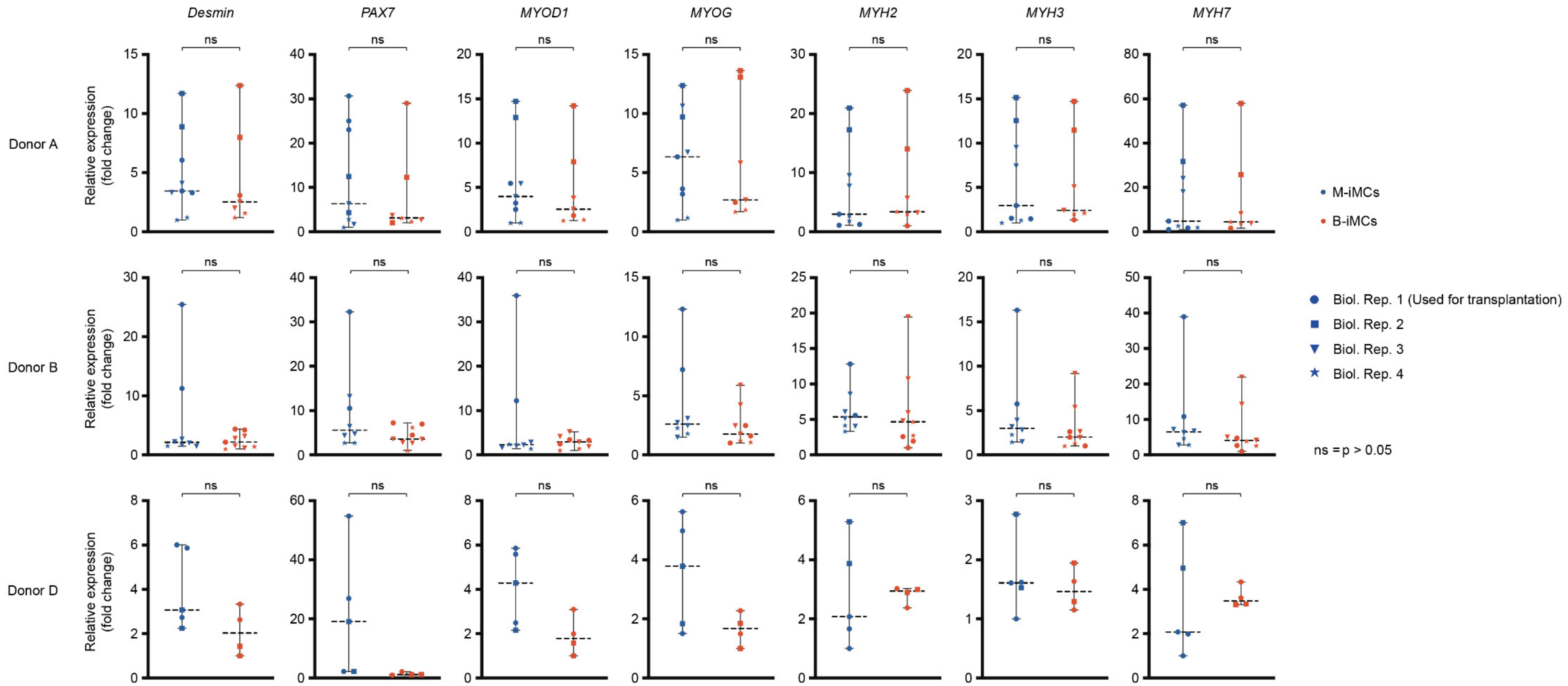

Figure S1. RT-qPCR quantification of the myogenic differentiation into M-iMPCs and B-iMPCs. Shown are the myogenic markers *Desmin, PAX7, MYOD1, MYOG,* and *MYH2/3/7* in iMPCs from donors A, B, and D differentiated from MiPS and BiPS after ~60 days of differentiation. ΔΔCt values are shown as fold change relative to the sample with the lowest expression value. Each dot represents a technical replicate (differentiation in an independent well). Donor A: n = 4; donor B: n = 3; donor D: n = 2 with duplicates for each experiment. Statistics: Student's t-test ($p < 0.05$); ns: not significant. Dashed lines represent the mean. Error bars in grey.

Figure S2

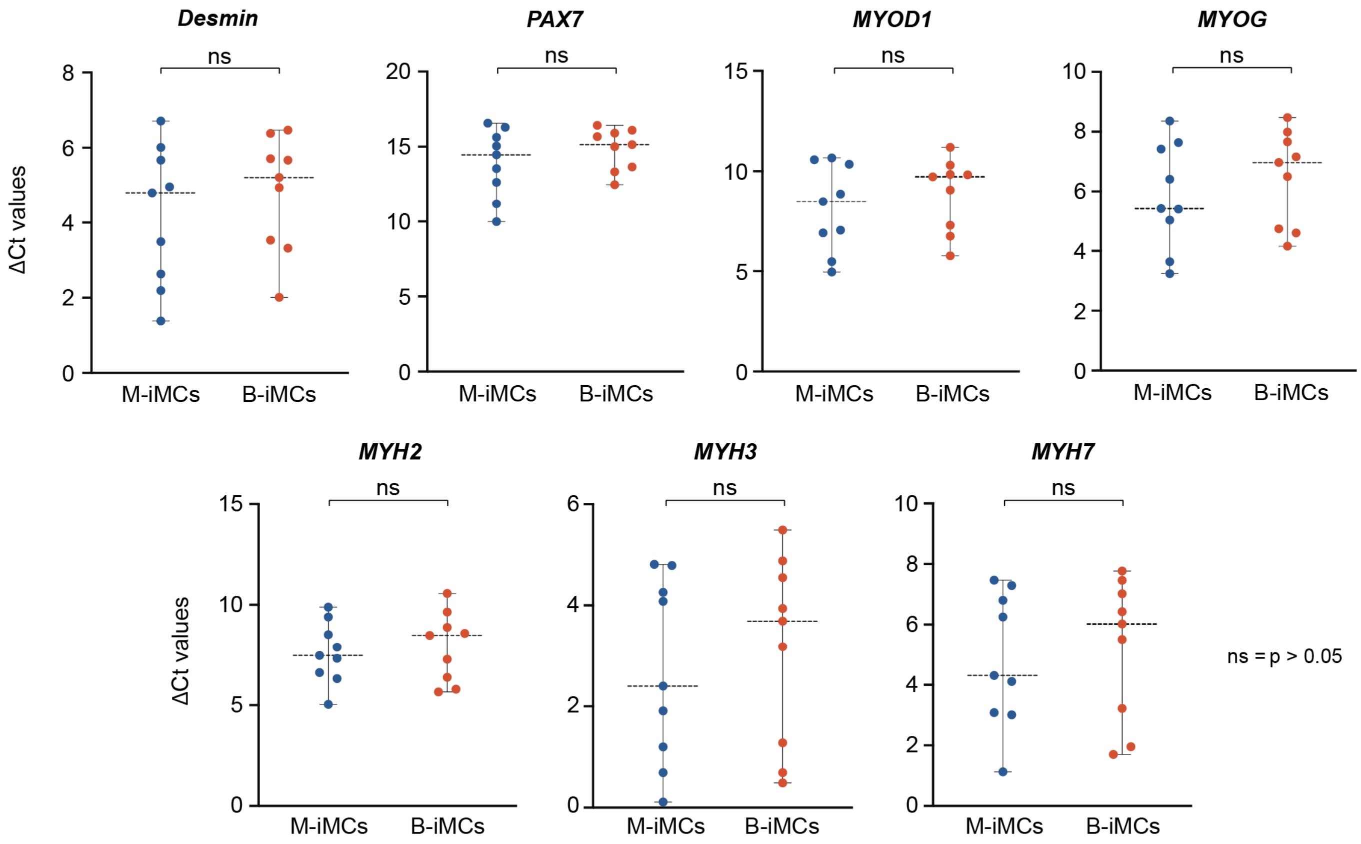

Figure S2. This figure shows the same data as Figure 3c but here shown as ΔCt values. RT-qPCR quantification from donors A, B, and D differentiated from MiPS and BiPS after ~60 days of differentiation. Statistics: Student's t-test ($p < 0.05$). Dashed lines represent the mean. Error bars in grey.

Figure S3

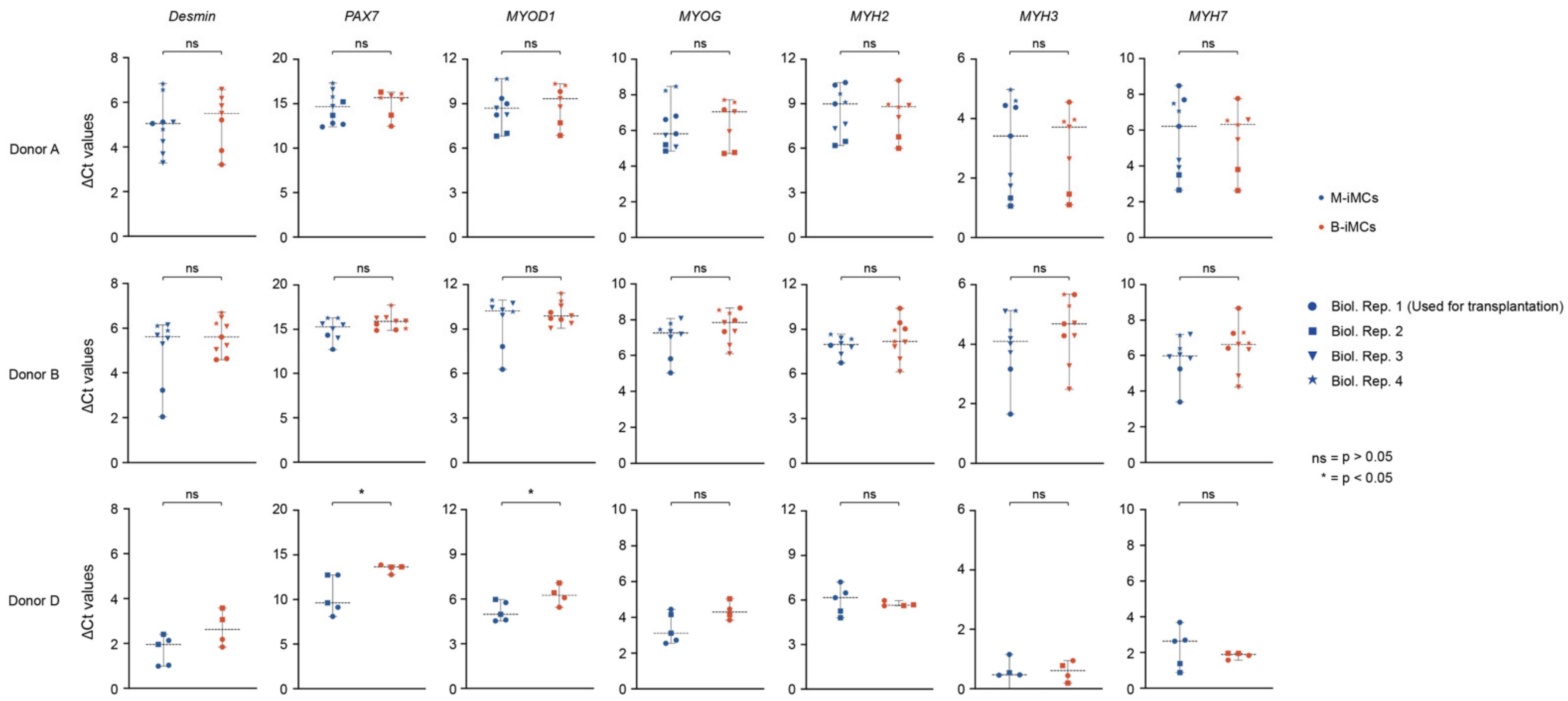

Figure S3. This figure shows the results from Supporting Information Figure S1 but here as ΔCt values. Each dot represents a technical replicate (differentiation in an independent well). Donor A: n = 4; donor B: n = 3; donor D: n = 2 with duplicates for each experiment. Statistics: Student's t-test ($p < 0.05$). Dashed lines represent the mean. Error bars in grey.

Figure S4

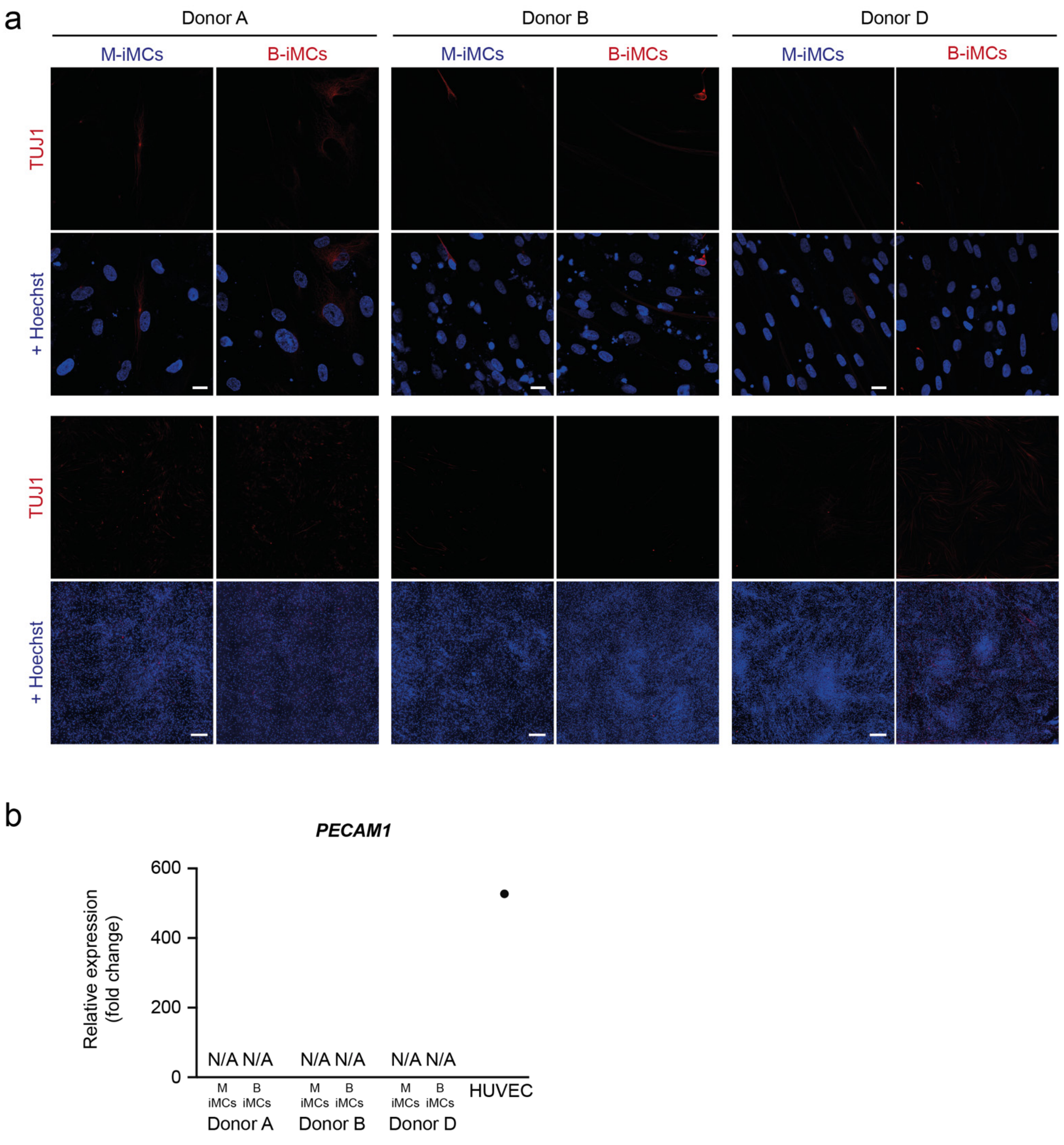

Figure S4. Absence of non-myogenic markers in iMCs after ~60 days of differentiation. (a) Immunofluorescence imaging of the neuronal marker class III beta tubulin (TUJ1). Nuclei: Hoechst. Upper panel: Scale bar: 20 µm. Lower panel: Scale bar: 500 µm. (b) RT-qPCR expression analysis of the endothelial marker PECAM1. ΔΔCt values are shown as fold change relative to the sample with the lowest expression value. All iMC samples exceeded Cts of 30 and melt curve analysis showed no PCR product for all iMC samples. HUVECs were used as control cell line. Statistics: Student's t-test ($p < 0.05$); ns: not significant. Dashed lines represent the mean.

Figure S5

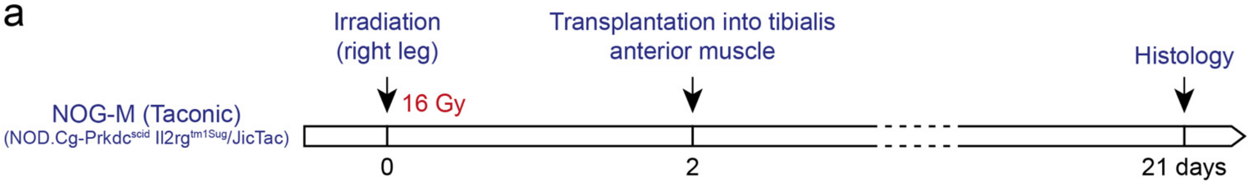

b

Transplanted cells		Successfull transplantations (human nuclei found)	Maximum number of human nuclei / mouse / cryosection	Human fibers per cryosection (Max/mouse)				PAX7+ nuclei per cryosection
				Mouse 1	Mouse 2	Mouse 3	Mouse 4	
Donor A	M-iMPCs	4/4	52 - 280	5	8	12	5	none found
	B-iMPCs	4/4	17 - 233	10	9	5	1	none found
Donor B	M-iMPCs	4/4	119 - 282	1	13	2	1	none found
	B-iMPCs	4/4	113 - 471	0	3	1	3	none found
Donor D	M-iMPCs	4/4	323 - 715	4	5	8	7	none found
	B-iMPCs	4/4	320 - 393	5	5	6	4	none found

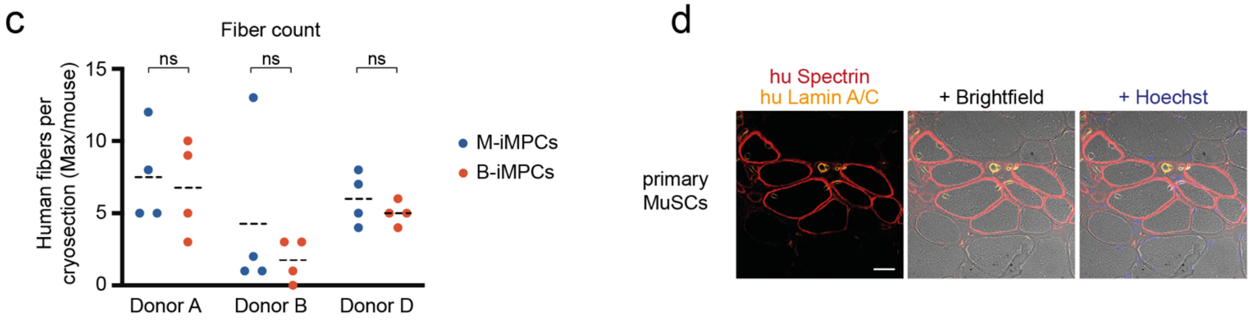

Figure S5. Cell transplantation into an immunocompromised mouse model. **(a)** Schematic description of the transplantation procedure: Focal 16 Gray irradiation of female 6–9-week-old xenograft-compatible NOG-M mice hind limb muscles (NOD.Cg-Prkdcscid Il2rgtm1Sug/JigTac) was performed using an image-guided robotic system. Two days after irradiation, freshly differentiated and dissociated iMPCs or control cells were injected into the central part of the tibialis anterior (TA) muscle. Then, 21 days after transplantation mice were sacrificed and muscles harvested. **(b)** List of transplantation results with numbers of successful transplantations (amount of engrafted mice), the maximum number of human nuclei found for one section within 4 mice (given are the numbers for the mouse with the lowest maximum and the highest maximum) and the maximum number of human fibers found in one section for each of the analyzed mice. **(c)** Quantification of human muscle fibers found in the transplants. The section containing the highest number of human myofibers in each transplant was counted. Statistics: Two-way ANOVA; ns: not significant. Dashed lines represent the mean. **(d)** Immunohistological analysis of the transplantation experiments using primary MuSCs. Shown are representative images of transversal tibialis anterior muscle sections of NOG-M mice. Sections are stained for human Spectrin, human Lamin A/C, and Hoechst. Scale bar: 20 µm.

Figure S6

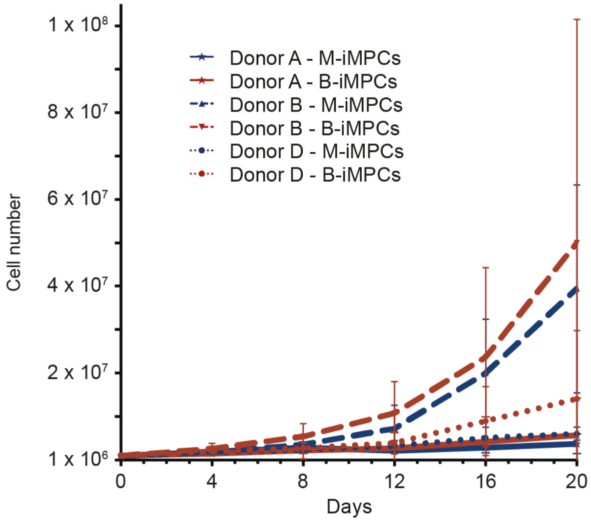

Figure S6. Proliferation curve of iMPCs after dissociation on day 30. Donor A: n = 4; donor B: n = 3; donor D: n = 2 with duplicates for each experiment. Error bars showing the mean.

Figure S7

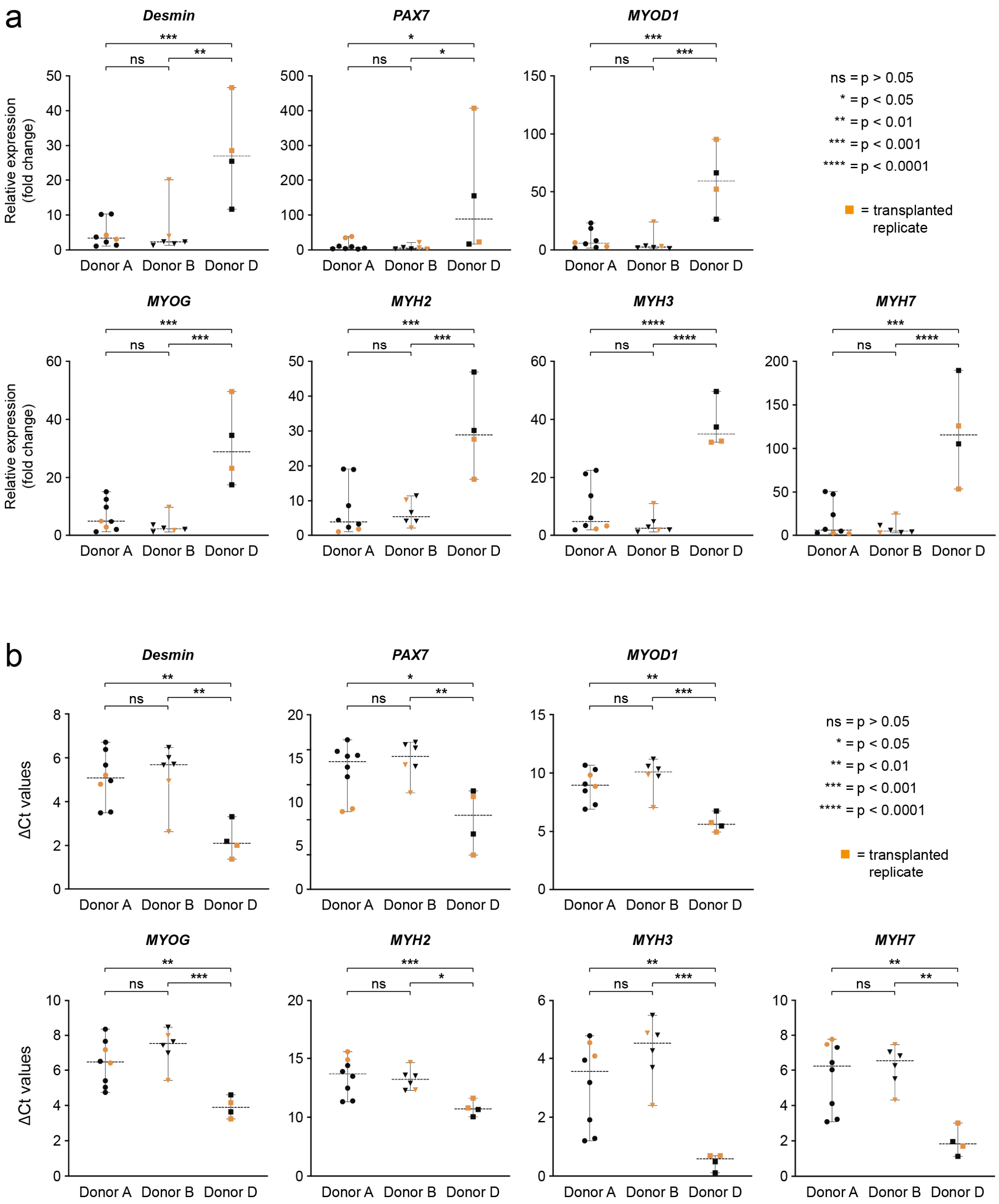

Figure S7. This figure shows the same results as Figure 5c. **(a)** The replicates that have been transplanted in the in vivo experiments are marked in orange. ΔΔCt values are shown as fold change relative to the sample with the lowest expression value. Donor A: n = 8; donor B: n = 6; donor D: n = 4. Each dot represents an independent experiment as mean of two technical replicates. Statistics: One-way ANOVA ($p < 0.05$). Dashed lines represent the mean. Error bars in grey. **(b)** Results are presented as ΔCt values. Donor A: n = 8; donor B: n = 6; donor D: n = 4. Each dot represents an independent experiment as mean of two technical replicates. Statistics: One-way ANOVA ($p < 0.05$). Dashed lines represent the mean. Error bars in grey.

Table S1. Primer list with annealing temperatures for RT-qPCR.

Gene		Primer	Annealing temperature
GAPDH	FWD	GAAGGTGAAGGTCGGAGTC	60 °C
	REV	GAAGATGGTGATGGGATTTC	
PAX7	FWD	TGGGCGACAAAGGGAA	60 °C
	REV	GGTAGTGGGTCCTCTCAAA	
MYOD1	FWD	GCGGAACTGCTACGAA	57 °C
	REV	AGATGCGCTCCACGAT	
Desmin	FWD	GGTACAAGTCGAAGGTGTCAG	60 °C
	REV	TCAATCTCGCAGGTGTAGGA	
MYOG	FWD	GCCAACCCAGGGGATCAT	60 °C
	REV	CCCGGCTTGGAAGACAATCT	
MYH2	FWD	GGAACGGGCTGACATTGCTG	60 °C
	REV	GTCATTCCATGGCATCAGGACA	
MYH3	FWD	GGAGCAGGACAGAAGATAT	55 °C
	REV	CCCAGATTGAAACAAAGCA	
MYH7	FWD	CTGTCCAAGTTCCGCAAGGT	60 °C
	REV	TCATTCAAGCCCTTCGTGCC	
PECAM1	FWD	TCGTGGTCAACATAACAGAACT	60 °C
	REV	TGAAGTTGGCTGGAGGTG	

Table S2. Reprogramming efficiencies and attempts for MuSCs and PBMCs.

		Number of appearing iPSC colonies		Number of reprogramming trials	
Donor	Sex/Age	MuSCs	PBMCs	MuSCs	PBMCs
A	Female, 47	38 (0.076%)	15 (0.005%)	1	1
B	Female, 50	42 (0.084%)	2 (0.0007%)	1	1
C	Male, 18	50 (0.1%)	5 (0.0016%)	1	5
D	Female, 47	36 (0.072%)	2 (0.0007%)	1	1
E	Male, 58	9 (0.018%)	0 (0%)	1	5
